# Sexual behaviour, STI and HIV testing and testing need among gay, bisexual and other men who have sex with men recruited for online surveys pre/post-COVID-19 restrictions in the UK

**DOI:** 10.1136/sextrans-2022-055689

**Published:** 2023-03-01

**Authors:** Jack RG Brown, David Reid, Alison R Howarth, Hamish Mohammed, John Saunders, Caisey V Pulford, Dana Ogaz, Gwenda Hughes, Catherine H Mercer

**Affiliations:** 1 Institute for Global Health, University College London, London, UK; 2 National Institute for Health Research Health Protection Research Unit in Blood Borne and Sexually Transmitted Infections at University College London in partnership with the UK Health Security Agency, London, UK; 3 Blood Safety, Hepatitis, STIs and HIV Division, UK Health Security Agency, London, UK; 4 Department of Infectious Disease Epidemiology, London School of Hygiene and Tropical Medicine, London, UK

**Keywords:** SEXUAL BEHAVIOUR, SEXUAL HEALTH, HIV, COVID-19, Sexual and Gender Minorities

## Abstract

**Objectives:**

We examined sexual behaviour, sexually transmitted infection (STI) and HIV testing and testing need, and identified associated factors, among gay, bisexual and other men who have sex with men (GBMSM) in the UK after COVID-19 restrictions ended, and compared these with ‘pre-pandemic’ estimates.

**Methods:**

We analysed survey data from GBMSM (N=1039) recruited via social media and Grindr in November–December 2021. We then compared Grindr-recruited 2021 participants (N=437) with those from an equivalent survey fielded in March–May 2017 (N=1902). Questions on sexual behaviour and service use had lookback periods of 3–4 months in both surveys. Unmet testing need was defined as reporting any new male and/or multiple condomless anal sex (CAS) partners without recent STI/HIV testing. Participants were UK residents, GBMSM, aged ≥16 years who reported sex with men in the last year. Multivariable logistic regression identified associated sociodemographic and health-related factors with unmet STI/HIV testing need in 2021, and then for 2017/2021 comparative analyses, adjusting for demographic differences.

**Results:**

In 2021, unmet STI and HIV testing need were greater among older GBMSM (aged ≥45 years vs 16–29 years; adjusted OR (aOR): 1.45 and aOR: 1.77, respectively), and lower for pre-exposure prophylaxis (PrEP) users (vs non-PrEP users; aOR: 0.32 and aOR: 0.23, respectively). Less unmet STI testing need was observed among HIV-positive participants (vs HIV-negative/unknown; aOR: 0.63), and trans and non-binary participants (vs cisgender male; aOR: 0.34). Between 2017 (reference) and 2021, reported sexual risk behaviours increased: ≥1 recent new male sex partner (72.1%–81.1%, aOR: 1.71) and ≥2 recent CAS partners (30.2%–48.5%, aOR: 2.22). Reporting recent STI testing was greater in 2021 (37.5%–42.6%, aOR: 1.34) but not recent HIV testing, and there was no significant change over time in unmet STI (39.2% vs 43.7%) and HIV (32.9% vs 39.0%) testing need.

**Discussion:**

Comparable community surveys suggest that UK resident GBMSM may have engaged in more sexual risk behaviours in late 2021 than pre-pandemic. While there was no evidence of reduced STI/HIV service access during this time, there remained considerable unmet STI/HIV testing need.

**WHAT IS ALREADY KNOWN ON THIS TOPIC**

- Sexual risk behaviour and access to sexual health services fluctuated over the COVID-19 pandemic, with some groups more adversely affected than others, so it is essential to monitor these following the lifting of social restrictions in summer 2021.

**WHAT THIS STUDY ADDS**

- After COVID-19 restrictions had ended, sexual risk behaviour and sexually transmitted infection (STI) testing—but not HIV testing—exceeded levels reported pre-pandemic.

**HOW THIS STUDY MIGHT AFFECT RESEARCH, PRACTICE OR POLICY**

- Similar levels of unmet STI/HIV testing need remain pre/post COVID-19 restrictions in the UK (corresponding to over one-third of GBMSM), which need addressing as sexual health services continue to reconfigure.

## Introduction

In the UK, gay, bisexual and other men who have sex with men (GBMSM) bear a disproportionate burden of sexually transmitted infections (STIs) including HIV. All sexually active GBMSM are advised to test for STIs and HIV annually.^1^ Those at increased risk of STIs and HIV, for example, those reporting multiple recent condomless anal sex (CAS) partners and HIV pre-exposure prophylaxis (PrEP) users (who are assumed to be engaging in greater risk behaviour), are recommended to test quarterly.^1^

On 23 March 2020, the UK entered its first national lockdown in response to the growing SARS-CoV-2 (COVID-19) pandemic.^2^ During this initial period of COVID-19-related social restrictions, sexual health services (SHS) had to rapidly reconfigure: in-person asymptomatic screening and walk-in appointments were suspended and patients directed online.^3^ Throughout 2020 and throughout 2021, varying degrees of social restrictions remained in place. Research suggests that behaviours with increased risk of STIs and HIV (hereon for brevity ‘sexual risk behaviour’), after an initial decrease during the first national lockdown, tended to fluctuate in line with social restrictions.^4 5^ National surveillance data on STI testing for the general population showed a 52%–59% decrease in testing in March/April 2020 (vs March/April 2019).^6^ Testing levels recovered as restrictions were eased and plateaued as restrictions were reintroduced in October 2020. A repeat cross-sectional, community-based survey of GBMSM also observed this trend of recovery and then plateau between March 2020 and April 2021 as social restrictions changed.^5^

On 19 July 2021, England lifted most social restrictions (with the other nations of the UK lifting their restrictions soon after).^7^ Changes included dropping mask mandates, removing restrictions on capacities in hospitality venues and on group socialisation, as well as the reopening of nightclubs. At that time, most UK adults had been offered a first (or second) dose of COVID-19 vaccine, to reduce the risk of serious illness.^8^ It is important to understand to what extent sexual risk behaviour changed in response to the high coverage of vaccination in the population and the lifting of social restrictions, and the extent to which SHS met STI and HIV testing need, especially given the context of rising COVID-19 infections and increasing pressure on health services in late 2021.^9^

We used data from a large, community-based cross-sectional survey among GBMSM in the UK conducted in November–December 2021 to estimate the prevalence of key sexual risk and STI and HIV testing behaviours in this population after social restrictions were lifted, and identify factors associated with unmet STI and HIV testing need. We then compared these findings with those from an equivalent 2017 survey to explore changes in behaviour and testing need pre-pandemic and after COVID-19-related social restrictions had ended.

## Methods

### Study design

The ‘Reducing inequalities in Sexual Health’ (RiiSH)-COVID surveys are repeat, cross-sectional online community surveys, each fielded for 2–3 weeks. Initially, they were rapidly deployed (based on a previous 2017 survey)^10 11^ to capture the effects of the UK’s first national lockdown on GBMSM’s sexual behaviour and health service use. The subsequent surveys (2–4) captured these effects at different stages of the UK’s COVID-19 pandemic response.^4 5^ The latest iteration, survey 4, captured the time after most COVID-19-related social restrictions had been lifted in the UK.

### Setting and sampling

#### 2021 survey

Participants were recruited from social networking sites (Facebook, Twitter and Instagram) and the geospatial dating application Grindr from 22 November to 12 December 2021. Adverts on these sites and applications directed individuals to the anonymous online survey. The first questions assessed eligibility, defined as: UK resident; aged ≥16 years; men (cis/transgender), trans women or gender-diverse people assigned male at birth (AMAB); reporting sex in the past year with a man (cis/transgender) or gender-diverse person AMAB. The survey took on average 10 min to complete. Online consent was obtained from all participants. No financial incentive was offered.

#### 2017 survey

Data from a comparable online survey of GBMSM are included in comparative analyses.^10^ The 2017 survey collected data in March–May 2017 and included UK resident men (cis/transgender), trans women or gender-diverse people AMAB; aged ≥16 years; reporting sex in the past year with a man (cis/transgender) or gender-diverse person AMAB. Participants were similarly recruited via social networking and dating platforms. The survey took on average 10 min to complete. Online consent was obtained from all participants. No financial incentive was offered. We describe this previous survey as the ‘2017 survey’ as distinct from the ‘2021 survey’.

### Data collection

The 2021 RiiSH-COVID survey was adapted from the 2017 survey^10 11^; the 2021 and 2017 surveys were administered using SNAPSurvey and Demographix software, respectively. Both surveys included comparable questions on STI/HIV testing, SHS use, sexual relationships and behaviour, use of drugs associated with chemsex (crystal methamphetamine, mephedrone or gamma-hydroxybutyrate/gamma-butyrolactone) and personal well-being (using Office for National Statistics’ well-being measures^12^). Additionally, the 2021 survey included questions on PrEP use and COVID-19 history (eg, infection, testing and self-reported symptoms). Questions about the last occurrence of behaviours and service use referred to a lookback period which related to around 3–4 months prior to each survey. This lookback period roughly corresponded to:

- 2021 survey: from early August 2021 until November/December 2021 (period after late July 2021, when most COVID-19 social restrictions had been lifted).
- 2017 survey: the 3 months prior to completion of the survey (March–May 2017).

### Data analysis

A total of 1039 unique participants completed the 2021 survey. The denominator for the HIV testing analyses was restricted to GBMSM reporting an HIV-negative/unknown status (N=919). Due to relatively small numbers of participants from ethnic minority groups, we grouped participants by whether they identified as white or not (hereafter: ‘all other ethnic groups’), similarly for gender minorities according to whether they identified as cisgender male or not (hereafter: ‘all other gender groups’). Our comparative analyses of the 2017 and 2021 surveys include only cis/trans GBMSM recruited via Grindr to ensure comparability between the two survey samples (2017: N=1902; 2021: N=437).

We used Stata (V.17) to calculate the percentage reporting key sociodemographic, health, sexual risk and STI/HIV testing behaviours. For the 2021 survey analyses, binary logistic regression was then used to examine associations between demographic, health and sexual risk behaviours and the primary outcome: unmet testing need (considered separately for STIs and HIV), defined as reporting one or more new male sex partners and/or multiple CAS partners in the lookback period without testing during that period.^1^

Explanatory variables that were statistically significant according to Pearson’s χ2 test (p<0.05) in binary regression were then included in multivariable logistic regression to identify independent associations with the primary outcome.

In the comparative analysis (2017 vs 2021), we compared sociodemographic profiles between the samples and used multivariable logistic regression to examine whether survey sample (2017 (reference category) vs 2021) was significantly associated with sexual behaviour, testing and testing need, adjusting for those sociodemographic characteristics where there were statistically significant differences (p<0.05) between the two samples.

## Results

### Participants’ characteristics (2021 survey)

Over half of the 1039 participants in the 2021 survey were recruited through social media (55.6%), with the remainder recruited from Grindr (online supplemental appendix 1). Participants had a median age of 41 years. The majority identified as cisgender (95.7%), white (88.1%), gay (80.9%), resided in England (85.6%), with around three-quarters (76.3%) born in the UK. More than half (56.8%) reported having a degree, and the majority (75.7%) reported some form of employment. Over one-third of participants (39.4%) lived alone and one-third (33.3%) lived with their partner(s). Around 1 in 10 participants (11.6%) reported living with HIV.

10.1136/sextrans-2022-055689.supp1 Supplementary data

### Sexual and risk behaviours since restrictions ended in 2021

**Figure 1:**
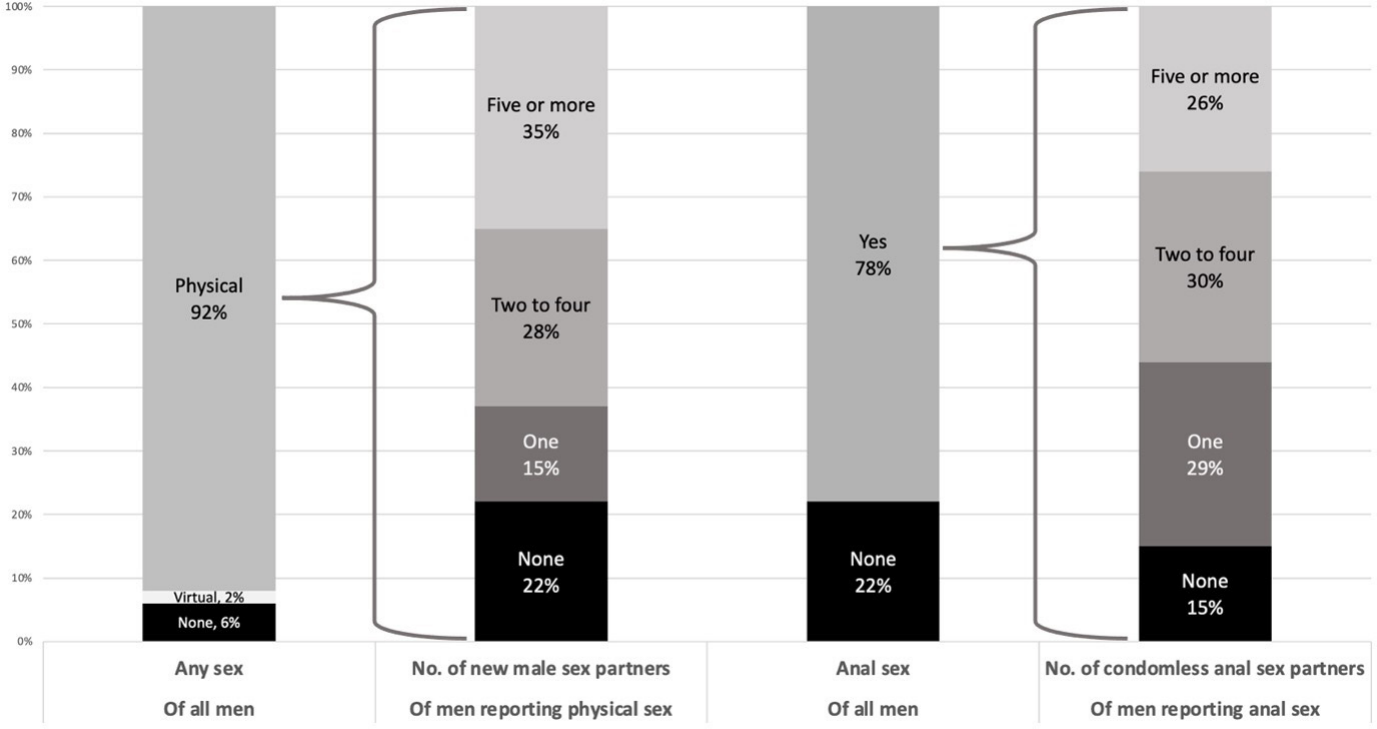
Sexual behaviour since COVID-19-related social restrictions lifted in the UK (early August–November/December 2021) reported by GBMSM participating in the 2021 RiiSH-COVID survey. GBMSM, gay, bisexual and other men who have sex with men; RiiSH, Reducing inequalities in Sexual Health.

Looking at reported sexual behaviour in the lookback period, 91.8% of participants reported physical sexual contact (defined as: any activity intended to achieve orgasm (or close to) for one or both partners), of whom 78.0% reported at least 1 new sex partner, 35.3% reported at least 5 and 18.7% at least 10 (figure 1 and online supplemental appendix 2). The most commonly reported ways of meeting new sex partners (of those reporting at least one) included: dating applications (77.8%), gay websites (41.1%) and cruising locations (21.0%).

Anal sex in the lookback period was reported by 78.3% of all participants; of those, 84.5% reported CAS with at least 1 partner, 55.3% reported CAS with multiple partners, 25.6% reported CAS with 5 or more partners, and 15.2% reported CAS with 10 or more. The use of chemsex drugs (crystal methamphetamine, mephedrone, gamma-hydroxybutyrate/gamma-butyrolactone) ever and in the lookback was reported by 14.4% and 5.5% of participants, respectively.

### SHS use, STI/HIV testing and testing need since restrictions ended in 2021

Visiting a sexual health clinic in the lookback was reported by 44.4% of participants (table 1). The most commonly reported reasons for doing so during the lookback were having a general sexual health check-up (56.1%), PrEP appointment (24.4%), and worrying about having an STI or HIV (17.2%). Altogether, 18.8% of participants reported STI symptoms in the lookback and 42.0% of all participants reported testing for STIs during this time, of whom 25.0% reported STI diagnosis/es. The most commonly reported ways of STI testing were at a sexual health clinic (60.0%) and via a free self-sample service (44.5%). Testing for HIV in the lookback was reported by 45.8% of HIV-negative/unknown participants, most commonly at a sexual health clinic (56.8%) or using a free self-sample service (34.2%; table 1).

**Table 1:** Sexual health service use since COVID-19-related social restrictions lifted in the UK (early August–November/December 2021) reported by GBMSM participating in the 2021 RiiSH-COVID survey

	% (n)
Visited a sexual health clinic	
Ever	81.0 (842)
In the lookback	44.4 (374)
Reason for most recent visit to sexual health clinic (if ever visited)	
General sexual health check-up	56.1 (471)
PrEP appointment	24.4 (205)
Worried might have STI or HIV	17.2 (144)
Symptomatic	13.6 (114)
Partner diagnosed with STI	9.8 (82)
HIV care	9.1 (76)
Vaccination	8.5 (71)
Partner symptomatic	5.6 (47)
PEP	5.6 (47)
Treatment after positive test	5.4 (45)
**STI-specific outcomes**	
STI symptoms (in the lookback)	
Yes	18.8 (195)
STI testing	
Ever	80.6 (837)
In the lookback	42.0 (436)
STI test result (if tested in lookback)	
Positive	25.0 (109)
Where tested for STIs (if tested in lookback)	
Sexual health clinic	60.0 (261)
Free self-sample service	44.5 (194)
HIV clinic	6.0 (26)
Private self-sample service	2.8 (12)
General practitioner	1.6 (7)
**HIV-specific outcomes (if reporting an HIV-negative/unknown status)**	
HIV testing	
Ever	88 (914)
In the lookback	45.8 (421)
Where tested for HIV (if tested in lookback)	
Sexual health clinic	56.8 (239)
Free online self-sampling	34.2 (144)
Self-testing kit	4.0 (17)
Another outpatient clinic	1.7 (7)
General practitioner	1.2 (5)
PrEP use	
In the lookback	31.9 (293)

GBMSM, gay, bisexual and other men who have sex with men; PEP, post-exposure prophylaxis; PrEP, pre-exposure prophylaxis; RiiSH, Reducing inequalities in Sexual Health; STI, sexually transmitted infection.

Around one-third of participants were considered as having had unmet testing need for STIs (35.7%) and HIV (32.1% among HIV-negative/unknown participants; table 2). Adjusting for factors associated with unmet testing need in the bivariable analysis, we found that unmet STI testing need in the lookback period was greater for older GBMSM (aged ≥45 years vs 16–29 years; adjusted OR (aOR): 1.45), and lower for trans/non-binary participants (vs cisgender male; aOR: 0.44), GBMSM living with HIV (vs being HIV-negative/unknown; aOR: 0.63) and PrEP users (vs non-PrEP users; aOR: 0.32). Similarly, among HIV-negative/unknown participants, unmet HIV testing need in the lookback period was greater for older GBMSM (≥45 years vs 16–29 years; aOR: 1.77), and lower for PrEP users (vs non-PrEP users; aOR: 0.23; table 2).

**Table 2:** Sociodemographic characteristics and health-related factors associated with unmet STI/HIV testing need since COVID-19-related social restrictions lifted in the UK (early August–November/December 2021) reported by GBMSM participating in 2021 RiiSH-COVID survey

	Unmet need for STI testing			Unmet need for HIV testing		
	% (n)	uOR (95% CI; p value)	aOR (95% CI; p value)*	% (n)	uOR (95% CI; p value)	aOR (95% CI; p value)†
All	35.7 (371)	–	–	32.1 (295)	–	–
**Sociodemographic**						
Age (years)		0.012	0.041		0.010	0.014
16–29	30.4 (69)	1	1	24.9 (55)	1	1
30–44	33.2 (128)	1.14 (0.80–1.62)	1.05 (0.73–1.51)	31.5 (105)	1.39 (0.95–2.04)	1.39 (0.94–2.06)
45 and over	40.9 (174)	1.59 (1.13–2.24)	1.45 (1.02–2.07)	37.0 (135)	1.77 (1.22–2.57)	1.77 (1.20–2.59)
Sexual identity		0.383	0.384		0.061	0.274
Gay	35.1 (295)	1	1	30.7 (225)	1	1
Bisexual‡	38.4 (76)	1.15 (0.84–1.59)	1.16 (0.83–1.63)	37.9 (69)	1.38 (0.98–1.94)	1.22 (0.86–1.73)
Gender identity		0.028	0.034		0.188	0.177
Cisgender male	36.4 (362)	1	1	32.5 (286)	1	1
All other gender groups§	20.0 (9)	0.44 (0.21–0.92)	0.44 (0.21–0.94)	22.5 (9)	0.60 (0.28–1.28)	0.59 (0.27–1.27)
Ethnicity		0.731	0.108		0.899	0.263
White	35.5 (325)	1	1	32.2 (259)	1	1
All other ethnic groups¶	37.1 (46)	1.07 (0.73–1.58)	1.41 (0.93–2.13)	31.6 (36)	0.97 (0.64–1.48)	1.30 (0.82–2.04)
Born in the UK		0.981	0.389		0.150	0.637
Yes	35.7 (283)	1	1	33.3 (234)	1	1
No	35.8 (88)	1.00 (0.74–1.35)	1.15 (0.84–1.57)	28.1 (61)	0.78 (0.56–1.09)	0.92 (0.64–1.31)
Residing in England		0.918	0.986		0.412	0.261
Yes	35.8 (318)	1	1	32.7 (253)	1	1
No	35.3 (53)	0.98 (0.68–1.41)	1.00 (0.69–1.46)	29.2 (42)	0.85 (0.58–1.25)	0.79 (0.53–1.19)
Highest educational qualification		0.727	0.599		0.137	0.561
Degree or higher	35.3 (208)	1	1	30.2 (162)	1	1
Below degree	36.3 (163)	1.05 (0.81–1.35)	0.93 (0.71–1.22)	34.8 (133)	1.24 (0.93–1.64)	1.09 (0.81–1.46)
**Health-related factors**						
HIV status		0.708	0.031			
Negative/unknown	35.9 (330)	1	1	–	–	–
Positive	34.2 (41)	0.93 (0.62–1.38)	0.63 (0.42–0.96)	–	–	–
PrEP use (in the lookback)		<0.001	<0.001		<0.001	<0.001
No	42.2 (315)	1	1	40.7 (255)	1	1
Yes	19.1 (56)	0.32 (0.23–0.45)	0.32 (0.23–0.45)	13.7 (40)	0.23 (0.16–0.33)	0.23 (0.16–0.33)
Life satisfaction level		0.492	0.595		0.342	0.530
Medium–very high	35.3 (302)	1	1	31.4 (237)	1	1
Low	38.0 (68)	1.12 (0.81–1.57)	1.10 (0.78–1.55)	35.2 (56)	1.19 (0.83–1.71)	1.13 (0.78–1.64)

*Adjusted for age, gender identity and PrEP use.

†Adjusted for age and PrEP use.

‡Including: bisexual (n=145), straight (n=6), other (n=47).

§Including: trans man (n=16), trans woman (n=3), other gender identities (n=26).

¶Including: black (n=16), Asian (n=58), mixed or other ethnic identities (n=50).

aOR, adjusted OR; GBMSM, gay, bisexual and other men who have sex with men; PrEP, pre-exposure prophylaxis; RiiSH, Reducing inequalities in Sexual Health; STI, sexually transmitted infection; uOR, unadjusted OR.

### Comparing behaviours reported pre/post-COVID-19 restrictions

Comparing estimates of behaviours reported by Grindr-recruited participants who completed the 2021 survey after COVID-19-related restrictions had been lifted with those reported by Grindr-recruited 2017 survey participants, 2021 participants were largely similar although slightly older (median age: 40 vs 37 years, respectively), less likely to report their ethnicity as white (84.9% vs 90.0%) and less likely to live in England (85.6% vs 93.7%) than other parts of the UK (online supplemental appendix 3).

We compared reporting of sexual behaviour, testing and testing need in the lookback period of the 2021 survey with that reported for the lookback of the 2017 survey (table 3). Adjusting for sociodemographic differences between the samples, participants in 2021 were more likely to report new male sex partners (aOR: 1.71), ≥5 new male sex partners (aOR: 1.71), ≥10 new male sex partners (aOR: 1.53), multiple recent CAS partners (aOR: 2.22), ≥5 recent CAS partners (aOR: 2.39), ≥10 recent CAS partners (aOR: 2.24) and recent STI testing (aOR: 1.34). Although reporting recent HIV testing was slightly higher in 2021 at 48.5% vs 45.5% in 2017, this was not a significant increase after adjusting for confounders. However, no significant difference was observed in the level of unmet need for either STI testing or HIV testing.

**Table 3:** Recent sexual behaviour, STI/HIV testing, and unmet testing need for STIs and HIV among GBMSM (cis/trans) recruited through Grindr, by data collection period (March–May 2017 (reference category) vs November–December 2021)

	2017 survey % (n)	2021 survey % (n)	uOR (95% CI)	P value	aOR (95% CI)*	P value
**Sexual behaviours, STI testing and testing need in the lookback period**						
≥1 new male sex partner(s)	72.1 (1127)	81.1 (351)	1.66 (1.27–2.16)	<0.001	1.71 (1.29–2.26)	<0.001
≥5 new male sex partners	26.3 (412)	36.3 (157)	1.59 (1.27–1.99)	<0.001	1.71 (1.35–2.16)	<0.001
≥10 new male sex partners	12.7 (198)	17.3 (75)	1.45 (1.08–1.93)	0.013	1.53 (1.13–2.07)	0.006
Anal sex	76.1 (1440)	80.3 (351)	1.28 (0.99–1.66)	0.060	1.35 (1.02–1.77)	0.033
Condomless anal sex (CAS)	54.3 (1032)	67.1 (293)	1.72 (1.38–2.14)	<0.001	1.83 (1.46–2.31)	<0.001
≥2 CAS partners	30.2 (563)	48.5 (212)	2.18 (1.76–2.69)	<0.001	2.22 (1.78–2.77)	<0.001
≥5 CAS partners	10.3 (192)	21.1 (92)	2.32 (1.77–3.06)	<0.001	2.39 (1.80 –3.17)	<0.001
≥10 CAS partners	5.6 (104)	11.4 (50)	2.19 (1.53–3.12)	<0.001	2.24 (1.56–3.23)	<0.001
STI test	37.5 (664)	42.6 (186)	1.23 (1.00–1.53)	0.053	1.34 (1.07–1.68)	0.010
Unmet need for STI testing†	39.2 (631)	43.7 (191)	1.20 (0.97–1.49)	0.088	1.12 (0.89–1.40)	0.335
**HIV testing and testing need of HIV-negative/untested participants in the lookback period**						
HIV test	45.5 (753)	48.5 (189)	1.13 (0.90–1.41)	0.287	1.25 (0.99–1.57)	0.065
Unmet need for HIV testing†	32.9 (494)	39.0 (152)	1.30 (1.03–1.64)	0.025	1.22 (0.96–1.56)	0.109

*Adjusted for age, gender identity, ethnicity and country of residence.

†Reported one or more new male sex partners and/or multiple CAS partners during lookback without reporting an STI/HIV test.

aOR, adjusted OR; GBMSM, gay, bisexual and other men who have sex with men; STI, sexually transmitted infection; uOR, unadjusted OR.

## Discussion

This latest large, community-based survey of GBMSM in the UK showed that the majority of participants reported sexual risk behaviours after the removal of COVID-19 social restrictions in 2021 and that this proportion was significantly higher than pre-pandemic (2017), for example, reporting ≥5 recent CAS partners doubled (21.1% vs 10.3%, respectively). In parallel, a greater proportion of participants in 2021 reported recent STI testing than in 2017, and there was no significant change between the two periods in the proportion with unmet testing need for STIs or HIV. However, over one-third of our 2021 sample had unmet testing need and this was more common among GBMSM aged 45 years and over.

### Comparison with other literature

There is little evidence available on GBMSM’s testing and risk behaviour since COVID-19-related social restrictions ended in the UK. However, research has shown that in the UK—and other European countries^13^—GBMSM’s sexual risk behaviour has tended to fluctuate in line with social restrictions, with sexual behaviour trending towards pre-pandemic levels as restrictions have eased.^5^ Our findings concur and suggest a large increase in sexual risk behaviours after social restrictions had ended. Furthermore, we observed that reported sexual risk behaviour in late 2021 not only returned to, but exceeded, pre-pandemic levels as reported in 2017. However, we should be mindful with this interpretation as we cannot account for increases in sexual risk behaviour independent of COVID-19 restrictions, as upward trends in sexual risk behaviour have been observed for some time; some attribute this to a widening use of dating applications to meet sex partners and the evidence of a greater confidence in HIV PrEP cited as drivers of this trend.^14^ However, another study of GBMSM, conducted around the same time as our 2017 baseline survey, found a similar proportion reporting multiple CAS partners in the previous 3 months.^15^

The proportion of participants reporting STI or HIV testing over time in previous RiiSH-COVID surveys has tended to follow testing trends observed by national surveillance systems, consisting of a rebound then plateau in the months following the first easing of social restrictions in June/July 2020.^5 6^ For example, HIV testing among RiiSH-COVID GBMSM with an HIV-negative/unknown status was reported by 29.7% of participants in a lookback period covering March–June/July 2020, before rising to 39.4% in July–November/December 2020 and levelling off at 40.5% in December 2020–March 2021.^5^ After restrictions were lifted in summer 2021, we observed an increase to 45.8% reporting recent HIV testing in August–November/December 2021. This level of HIV testing was not significantly higher than in the 2017 survey, although we did not find this for STI testing, where there was a significant increase in the proportion reporting a recent test in 2021 vs 2017. It is interesting to note that HIV testing has not increased in line with STI testing; this could be due to an expanded remote testing service during and after COVID-19,^16^ which yields a lower return rate for HIV than STIs due to self-finger prick blood test.^17^ Routinely commissioned PrEP has also become more readily available and widespread,^18^ which could lead to percieving a lesser need for HIV testing and thus just testing for STIs. However, it seems that the levels of testing observed (despite a non-significant increase in HIV testing) were sufficient to not observe a significant increase in unmet testing need.

### Strengths and limitations

We used the same study protocol and similar recruitment methods for this RiiSH-COVID survey and an earlier survey undertaken in 2017, resulting in large samples with broadly comparable sociodemographic profiles, and enabling our pre/post-COVID-19 comparison.^10^ Our findings also complement national surveillance data on SHS attendees by providing community-recruited samples of GBMSM thereby enabling comparisons on risk behaviours and testing need in GBMSM who do and do not access SHS.

However, there are limitations. As a cross-sectional survey, associations between variables can be bidirectional and therefore we cannot infer causality. We also acknowledge the issue of temporality in participants’ behaviours, for example, we cannot determine whether the behaviour occurred before or after reporting testing and thus this uncertainty goes into our measures of unmet need. Our comparison of the late 2021 and 2017 surveys may not be truly reflective of the impact of COVID-19-related social restrictions on behaviours, as we cannot account for changes that occurred independently of restrictions. Although these two surveys did follow the same protocol and may well be indicative of trends to compliment comparisons of national surveillance data comparing 2019 and 2021, we unfortunately did not have survey data from 2019.

Recruitment through an online survey and through social media and dating applications will exclude GBMSM who do not use these platforms, are not seeking new sexual partners, and/or do not have internet access, limiting the generalisability of our findings to all GBMSM. Given the small number of migrants and participants from ethnic minority groups (despite attempts to boost the number of participants from these groups in previous surveys by using different images and social media platforms to promote our survey), we needed to categorise country of birth and ethnicity as binary variables thereby overlooking substantive differences in sexual health within these groups.^10 19^ Likewise, as the majority of participants were cisgender GBMSM, we were unable to make meaningful inferences on barriers to access, and the sexual health needs of gender minorities.^20 21^ The variables we derived to try and capture unmet STI/HIV testing need were informed by national guidelines,^1^ which advise quarterly STI and HIV testing by GBMSM engaging in certain risk behaviours. We acknowledge that this is a crude measure and does not take account of subjective risk and the impact this has on a participant’s perception of ‘needing’ to test; for example, participants need for an HIV test if their partner has an undetectable HIV viral load^22^ or if they are using PrEP as guided, but we were unable to measure this in our surveys.

### Implications

This study provides vital information to support public health messaging, future public health planning and efforts to mitigate against risk to health if COVID-19-related restrictions were to be reinforced in the future. It also helps give context to GBMSM’s risk behaviours after restrictions had been eased to help inform responses and modelling efforts around emerging infectious outbreaks specific to this population, for example, the Mpox (monkeypox) outbreak among GBMSM in the UK in spring/summer 2022.^23^

It is encouraging to note that there is no significant difference in unmet testing need between pre-COVID-19 and post-COVID-19 restrictions, at least as captured by the data presented in this paper. However, the relatively high proportion of GBMSM considered to have unmet STI/HIV testing need throughout and following the periods of social restrictions and reconfiguration of SHS provision should be of concern to sexual healthcare professionals and policymakers, particularly for those GBMSM aged 45 years and older. As services continue to reconfigure, and the modes of testing for STIs and HIV shift, there may be a need to target those with greater unmet testing need to ensure equity of access. Protocols that signpost asymptomatic patients to online and remote testing may enable those with greater need to access in-person testing and services—and more promptly. Education and attitudinal interventions around accessing and using online services may be warranted to increase uptake, especially among those with less experience of, or preference for, accessing care in this way. This could help prevent a widening of digital inequalities and barriers that some groups may have experienced, and continue to experience, due to the rapid shifts to online testing pathways and SHS access that were necessary during the COVID-19 pandemic.

## Data Availability

Data are available upon reasonable request.
