# Supplementary material for: Sexual behaviour, STI and HIV testing and testing need among gay, bisexual and other men who have sex with men recruited for online surveys pre/post-COVID-19 restrictions in the UK
Source: Sex Transm Infect. 2023 Mar 1;99(7):467–73. doi: 10.1136/sextrans-2022-055689 (PMC10715464; doi:10.1136/sextrans-2022-055689)
Supplement: Supplementary data [file sextrans-2022-055689supp001.pdf]

Appendix 1: Characteristics of GBMSM participating in the 2021 RiiSH-COVID survey by recruitment site

	**2021 All participants (N= 1,039) % (n)**	**2021 participants recruited through social media (N=567) % (n)**	**2021 participants recruited through Grindr (N=452) % (n)**	**p-value** *for differences in samples by recruitment site*
**Participants' characteristics**				
**Median Age (years) (IQR; range)**	41 (31-51; 17-81)	42 (31-52; 17-81)	39 (31-50; 17-77)	
**Age category (years)**				
16-29	21.9 (227)	21.7 (123)	22.8 (103)	0.024
30-44	37.2 (386)	33.9 (192)	40.9 (185)	
45 and over	40.9 (425)	44.4 (251)	36.3 (164)	
**Gender identity**				
Cis male	95.7 (994)	96.1 (545)	95.1 (430)	0.671
Trans male	1.5 (16)	1.6 (9)	1.6 (7)	
Trans female	0.3 (3)	0.4 (2)	0.2 (1)	
All other gender identities	2.5 (26)	1.9 (11)	3.1 (14)	
**Ethnicity**				
White (inc. white minorities)	88.1 (915)	90.5 (513)	84.7 (383)	0.005
Black	1.5 (16)	0.5 (3)	2.9 (13)	
Asian	5.6 (58)	4.6 (26)	7.1 (32)	
Mixed and/or other ethnic groups	4.8 (50)	4.4 (25)	5.3 (24)	
**Sexual identity**				
Gay	80.9 (841)	86.2 (489)	74.6 (337)	<0.001
Bisexual	14.0 (145)	9.9 (56)	19.3 (87)	
Straight	0.6 (6)	0.7 (4)	0.4 (2)	
All other sexual identities	4.5 (47)	3.2 (18)	5.8 (26)	
**Country of residence in the UK**				

England	85.6 (889)	85.7 (486)	85.2 (385)	0.098
Scotland	7.5 (78)	6.9 (39)	8.6 (39)	
Wales	4.7 (49)	5.8 (33)	3.3 (15)	
Northern Ireland	2.2 (23)	1.6 (9)	2.9 (13)	
**Born in the UK**				
Yes	76.3 (793)	77.1 (437)	75.4 (341)	0.543
**Highest educational qualification**				
Degree or higher	56.8 (590)	59.1 (335)	53.5 (242)	0.076
**Currently employed**				
Yes	75.7 (786)	76.7 (435)	75.0 (339)	0.523
**Living alone**				
Yes	39.4 (409)	35.8 (203)	43.1 (195)	0.017
**Living with partner(s)**				
Yes	33.3 (346)	38.3 (217)	26.6 (120)	<0.001
**Anxiety level**				
High	36.1 (372)	35.7 (202)	36.3 (162)	0.835
**Life satisfaction level**				
Low	17.3 (179)	15.7 (89)	19.4 (87)	0.120
**Living with a HIV diagnosis**				
Yes	11.6 (120)	12.2 (69)	10.6 (48)	0.441
**PrEP use (in the lookback) if HIV-negative/unknown**				
Yes	31.9 (293)	29.3 (146)	34.4 (139)	0.102

Appendix 2: Sexual behaviour reported since restrictions lifted (August-November/December 2020) by GBMSM participating in the 2021 RiiSH-COVID survey

	**2021 survey (N=1,039)**
**Sexual behaviour (reported in the lookback)**	
**No. of new partners (if reporting sex)**	N=946
None	22.0 (208)
One	14.7 (139)
2-4	28.0 (265)
5-9	16.6 (157)
10 or more	18.7 (177)
**Where participants met new sex partners**	N=738
Dating applications	77.8 (574)
Gay websites	41.1 (303)
Cruising location	21.0 (155)
Private gay sex party	10.7 (79)
Through friends	14.5 (107)
Within household	11.5 (85)
Elsewhere	18.6 (137)
Through gay organisation or group	7.3 (54)
**No. of CAS partners (if reporting any CAS)**	N=688
One	34.6 (238)
2-4	35.2 (242)
5-9	12.2 (84)
10+	18.0 (124)
**Use of chemsex drugs**	N=1039
Ever	14.4 (150)
In the lookback	5.5 (57)

Appendix 3: Characteristics of cis/trans identifying GBMSM recruited via Grindr for the 2017 and 2021 RiiSH surveys.

	**2021 Grindr sample (N=437) % (n)**	**2017 Grindr sample (N=1902) % (n)**	**P value (differences between profiles in 2021 vs 2017)**
**Demographics**			
	**Median (IQR: range)**	**Median (IQR: range)**	
**Age (years)**	40 (31-50; 17-77)	37 (28-48; 16-76)	
16-29	22.7 (99)	29.8 (566)	0.009
30-44	40.7 (178)	37.5 (713)	
45-59	29.3 (128)	29.3 (128)	
60+	7.3 (32)	7.3 (32)	
**Ethnic group**			
White	84.9 (371)	90.1 (1688)	0.011
Black	2.8 (12)	2.2 (42)	
Asian	6.9 (30)	3.8 (72)	
Mixed or other	5.5 (24)	3.8 (72)	
**Gender identity**			
Cisgender male	98.4 (430)	99.4 (1890)	0.041
Transgender male	1.6 (7)	0.6 (12)	
**Country of residence**			
England	85.4 (373)	93.7 (1782)	<0.001
Scotland	8.7 (38)	0.6 (12)	
Wales	3.2 (14)	5.6 (106)	
Northern Ireland	2.8 (12)	0.1 (2)	
**Educated to degree level**	53.8 (235)	50.4 (951)	0.203
**Single**	63.8 (279)	61.9 (1173)	0.450
**HIV positive**	10.8 (47)	11.4 (217)	0.697
